# Exploring the Role of Complexity in Health Care Technology Bottom-Up Innovations: Multiple-Case Study Using the Nonadoption, Abandonment, Scale-Up, Spread, and Sustainability Complexity Assessment Tool

**DOI:** 10.2196/50889

**Published:** 2024-04-26

**Authors:** Ulla Hellstrand Tang, Frida Smith, Ulla Leyla Karilampi, Andreas Gremyr

**Affiliations:** 1 Department of Prosthetics and Orthotics, Sahlgrenska University Hospital Gothenburg Sweden; 2 Department of Orthopaedics, Institute of Clinical Sciences, Sahlgrenska Academy at University of Gothenburg Gothenburg Sweden; 3 Regional Cancer Centre West Gothenburg Sweden; 4 Department of Technology Management and Economics, Collaborative Plattform for Healthcare Improvement, Chalmers University of Technology Gothenburg Sweden; 5 Department of Schizophrenia Spectrum Disorders, Sahlgrenska University Hospital Gothenburg Sweden; 6 Jönköping Academy for Improvement of Health and Welfare, School of Health and Welfare, Jönköping University Jönköping Sweden

**Keywords:** digital, bottom-up innovation, complexity, eHealth, health care, nonadoption, abandonment, scale-up, spread, and sustainability complexity assessment tool, NASSS-CAT, mobile phone

## Abstract

**Background:**

New digital technology presents new challenges to health care on multiple levels. There are calls for further research that considers the complex factors related to digital innovations in complex health care settings to bridge the gap when moving from linear, logistic research to embracing and testing the concept of complexity. The nonadoption, abandonment, scale-up, spread, and sustainability (NASSS) framework was developed to help study complexity in digital innovations.

**Objective:**

This study aims to investigate the role of complexity in the development and deployment of innovations by retrospectively assessing challenges to 4 digital health care innovations initiated from the bottom up.

**Methods:**

A multicase retrospective, deductive, and explorative analysis using the NASSS complexity assessment tool LONG was conducted. In total, 4 bottom-up innovations developed in Region Västra Götaland in Sweden were explored and compared to identify unique and shared complexity-related challenges.

**Results:**

The analysis resulted in joint insights and individual learning. Overall, the complexity was mostly found outside the actual innovation; more specifically, it related to the organization’s readiness to integrate new innovations, how to manage and maintain innovations, and how to finance them. The NASSS framework sheds light on various perspectives that can either facilitate or hinder the adoption, scale-up, and spread of technological innovations. In the domain of condition or diagnosis, a well-informed understanding of the complexity related to the condition or illness (diabetes, cancer, bipolar disorders, and schizophrenia disorders) is of great importance for the innovation. The value proposition needs to be clearly described early to enable an understanding of costs and outcomes. The questions in the NASSS complexity assessment tool LONG were sometimes difficult to comprehend, not only from a language perspective but also due to a lack of understanding of the surrounding organization’s system and its setting.

**Conclusions:**

Even when bottom-up innovations arise within the same support organization, the complexity can vary based on the developmental phase and the unique characteristics of each project. Identifying, defining, and understanding complexity may not solve the issues but substantially improves the prospects for successful deployment. Successful innovation within complex organizations necessitates an adaptive leadership and structures to surmount cultural resistance and organizational impediments. A rigid, linear, and stepwise approach risks disregarding interconnected variables and dependencies, leading to suboptimal outcomes. Success lies in embracing the complexity with its uncertainty, nurturing creativity, and adopting a nonlinear methodology that accommodates the iterative nature of innovation processes within complex organizations.

## Introduction

### Why Is it so Difficult to Develop and Spread New Innovative Technologies in Health Care?

There has been an increasing focus on innovation and the role of new technologies (eg, electronic health records, smartphones, and health applications) in health care. However, developing new technologies comes with significant challenges. Studies show that technology projects in health care, particularly large and complex projects, have a high rate of failure and seldom produce the anticipated results [1-5]. Bottom-up innovations in health care are innovations for service delivery that have been developed “from the ground up,” often focusing on preventive patient-centered care, typically driven forward by small interdisciplinary groups of professionals and patients [6-9]. As a result, they may not be captured by existing metrics, thus being “invisible” to senior management and policy makers [10].

The challenges when it comes to developing and making use of innovations, such as spreading or implementing new ways of working [11], have been described by many, often as a “knowledge translation or production problem” [12]. Braithwaite et al [13] compared the traditionally dominant linear and causal thinking that characterizes early implementation science and the evidence-based medicine paradigm with features such as those in systems thinking. The linear approach applies simple, orderly processes with cumulative sequences of stages to produce results building on a knowledge of the way things work, making use of predictable relationships between causes and effects. This has helped generate many successes in the past, not least in health care, but it tends to increase rigidity and fail when applied in more complex and messy systems where things change dynamically and are therefore unpredictable [13]. Instead, systems thinking and the related complexity science recognize system characteristics in building an understanding of how best to move forward.

To drive change in a predictable “simple” system where causes and effects are known, a linear, stepwise approach has a greater chance of success. However, complex systems are not only dynamic but also are often described as adaptive (as in complex adaptive systems) in that they are constituted of agents and artifacts that communicate and learn from each other and the surrounding environment, creating opportunities to learn from experience, self-organize, and evolve, making them less predictable systems [14].

Even though the linear approach has previously dominated implementation and development initiatives in health care, many researchers point to the necessity to apply systems thinking and complexity science when developing health care through the innovative use of new technologies, as exemplified in the study by Greenhalgh and Papoutsi [2]. As the concept of stepwise, linear cause and effect is not sufficient when studying complex systems that evolve in ways that are impossible to predict, it is relevant to use the knowledge of complex systems when understanding and studying health services [15]. Complex systems are defined by (1) intricate intertwined processes, (2) interconnectivity between systems, (3) interconnectivity between levels within systems, and (4) interconnectivity between actors and elements, giving complex systems different properties from those of less complex systems [1]. In short, a complex system does not work linearly but dynamically, with fundamentally different logics [16], and needs to be addressed and understood accordingly during innovation and implementation. If not, there is a risk that new technology and innovations will further increase the complexity rather than actually supporting the needs and demands for an improved health care system [17].

### The Challenges

A total of 4 bottom-up innovators found that there was a need to gain insights into the complexity involved in developing and executing bottom-up innovations in a complex health care organization. All 4 innovators had met with hindrances preventing them from moving forward with their innovations. It was necessary to pause and retrospectively try to comprehend the underlying reasons for the stagnation in the 4 cases in question.

Project representatives, all health care professionals, joined forces to identify challenges by assessing project complexity to increase an understanding of the role of complexity and find ways to explore and assess it. As they all worked within the same regional system, it was crucial to involve regional stakeholders (support functions) during the learning process.

The aim of this study was to investigate the role of complexity in the development and deployment of innovations by retrospectively assessing the challenges to 4 digital health care innovations initiated from the bottom up.

## Methods

This section describes the theoretical framework that underlies our methodological approach, the settings, and the 4 cases under study, as well as the procedure.

### Theoretical Framework

An impressive amount of knowledge related to the diffusion of innovations and their implementation in health care by the start of the new millennium is summarized in the extensive review by Greenhalgh et al [11] from 2004. It builds partly on the ideas by Rogers [18] that innovations have characteristics that will affect their diffusion, as well as affect other domains (eg, the readiness of the system for change, the implementation process, the adopter, and the external wider [sociopolitical] context). As innovations in health care were increasingly associated with new technologies, a new review was conducted by Robert et al [19] in 2010, adding more recent data and focusing on the adoption and assimilation of new technologies into health care.

**Figure 1:**
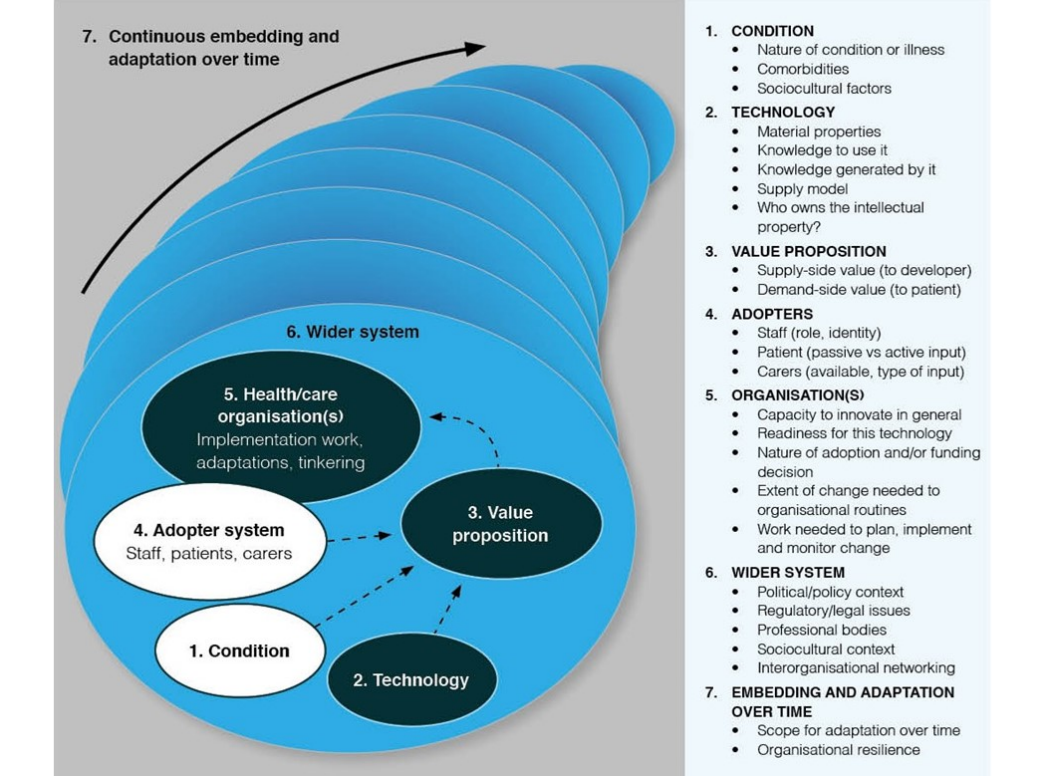
The nonadoption, abandonment, scale-up, spread, and sustainability complexity assessment tool with its 7 domains.

This, along with the high failure rate of health care technology innovation projects, inspired Greenhalgh and colleagues to deepen their knowledge of the diffusion of innovations, with an emphasis on health technology projects. Building on previous work, reviewing the literature, and using empirical studies of technology implementation, they elaborated on and explained domains of importance. This resulted in the nonadoption, abandonment, scale-up, spread, and sustainability (NASSS) framework (Figure 1 [20], published under Creative Commons Attribution 4.0 International License, CC BY).

The NASSS framework was developed into a complexity assessment tool (NASSS-complexity assessment tool [NASSS-CAT]) [20] to help assess the complexity of health technology projects before, during, or after they were finished.

### Design and Methodological Approach

**Figure 2:**
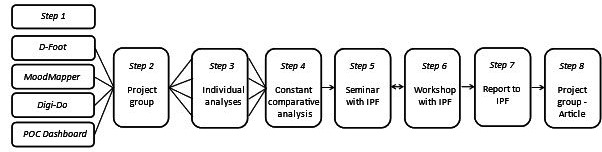
Flowchart illustrating the study process when exploring the role of complexity in health care technology projects. This figure presents the different steps (1-8) in the study. Details can be found in the main text. IPF: Innovation Platform.

A multicase retrospective, deductive, and explorative analysis using the NASSS-CAT LONG was conducted [15,21]. The process of analysis is shown in Figure 2 and is described at the end of the *Methods* section. The complexities of 4 bottom-up innovations developed in Region Västra Götaland (VGR) in Sweden were explored. The NASSS-CAT LONG consists of 2 parts divided into 7 domains (Figure 1). First, one is asked to describe the project and its potential messiness in their own words. Writing this narrative can help surface interdependencies and tricky issues of the project, hence revealing complexity. Second, one answers the questions related to the domain to help them estimate key areas of complexity. One can define whether the question is complex or not complex, whether they do not know, or whether it is not applicable. The total score of orange boxes ticked tells one how complex a certain domain is for their project. In part 2, one is guided through prompts to help them plan for and manage complexity by *reducing* it where possible and *responding* to it if or where it cannot be reduced. The questions can be answered by different people who will provide the needed insights into the domain and the project under evaluation.

### Setting

The 4 bottom-up innovators were from different parts of the organization in the VGR [22]. All of them had unique experiences of their own departments of the VGR and the surrounding supporting systems, such as the IT departments, purchasing departments, and legal offices. Although all 4 worked in specialized care, the care flow for each of the relevant diagnoses spanned specialized care, primary care, and municipal care.

The VGR is a region on the western coast of Sweden with a total population of 1.8 million. For several years, there has been a push for increasing the development of innovative solutions to the challenges faced by, for example, the public health care system. This has been implemented through the formation of an Innovation Platform (IPF) that develops processes and supportive structures as well as approving funds to help innovative ideas thrive. The mission of the IPF is to contribute to a sustainable innovation system that promotes innovation in health care and ensures that collaboration between academia and business fulfills the needs of patients within the health care system.

In total, 4 bottom-up innovators in the VGR pioneering eHealth and clinical research united in 2019 after realizing that there were barriers to their separate innovations due to an organizational lack of an innovation framework and to inexperience in developing, testing, implementing, and maintaining digital innovations. One author, familiar with the NASSS framework, encouraged the others to explore complexity in innovation. Together, they adapted the NASSS-CAT to identify unique and shared complexities in their innovations. Concurrently, at the same network meeting, representatives from the IPF wanted to be a part of the study, exchanging insights on how to identify and manage complexities in the innovation process.

### Ethical Considerations

Because no personal data were collected, no ethics approval was needed. All participants agreed, orally, to take part. No sensitive personal information was collected, and no patients participated in the workshops. The IPF, the regional support resource for innovations in health care, read and commented on the Swedish report before publication.

### The 4 Bottom-Up Innovations

#### Overview

Each innovation is presented in the following sections and in Multimedia Appendix 1 [15,20,23-38]. The cases are heterogeneous with regard to intended user, phase in the innovation process, and place of implementation (locally, regionally, and nationally). Despite their differences, they all existed in the same environment, framed by the regulations of the VGR and its support system for innovation.

#### Case 1: The D-Foot

The lifetime risk of developing diabetic foot ulcers (DFUs) is as high as 34% for patients with diabetes [23]. It is a burden for the patient and for health care with regard to costs. With prompt prevention, the prevalence of DFUs can be halved [24].

The D-Foot is a digital decision support system designed for preventing DFUs. It conducts early screening and provides treatment recommendations based on a risk grade (ranging from 1=no risk to 4=ongoing foot ulcers) [25,26]. The risk grade is automatically generated through a series of structured foot assessments and patient surveys [27]. A printable report of foot assessments, risk grade, and recommendations is generated. The innovation’s reliability and usability have previously been reported and assessed as good [27,28]. The innovator’s intention was that the D-Foot would serve as a tool in the national effort to implement a person-centered and seamless care chain for preventing foot ulcers in people living with diabetes [25,26].

The seamless care chain consists of (1) an annual foot examination, (2) a podiatry intervention, (3) the provision of appropriate footwear for at-risk patients, and (4) treatment in a multidisciplinary team for patients with active DFUs [25,39]. The D-Foot was developed as an easy-to-use digital tool to support foot examinations for individuals diagnosed with diabetes, primarily targeting prosthetic and orthotic specialist care [27,28,40]. The goal was to implement the D-Foot nationally, expecting early prevention of DFUs, improved quality of life for affected individuals [29,41], and reduced health care costs [42].

Version 1.0 of the D-Foot software was developed from 2011 to 2016 by an expert group comprising certified prosthetists and orthotists, patient representatives, and orthopedic surgeons in the VGR [27]. Initially, it underwent regional testing with positive results. Thereafter, continuous improvements have been made based on users’ comments [28]. Not yet executed is the request from users for integration between the D-Foot and the major medical record system [28].

#### Case 2: The MoodMapper

Bipolar disorder, often diagnosed in early adulthood, typically necessitates lifelong treatment. It leads to undesirable mood swings affecting daily functioning. Mood episodes vary from extreme “highs” (manic episodes) to severe “lows” (depressive episodes) lasting for days or weeks. Even with proper treatment, mood fluctuations can occur. Collaborative communication between patients and health care providers enhances treatment effectiveness. Moreover, the early detection of behavior changes is of the utmost importance in the successful treatment of bipolar disorder.

The aim of this innovation was to determine whether smartphone use data are a reliable source for studying changes in the digital behavioral patterns of individuals with bipolar disorder by exploring correlations between different parameters of smartphone use data.

The MoodMapper is a mobile app that, through real-time data collection, can provide valuable insights into a patient’s smartphone use. The ambition was, after pilot-testing, to study and evaluate the connection between mobile-generated passive data and documented changes in the patient’s mental well-being, with the goal of making it easier for both patients and health care professionals to monitor the progression of the patient’s condition and make decisions regarding prevention and care.

#### Case 3: The Digi-Do

Radiation therapy (RT) is a common treatment after breast cancer surgery. The high-technology environment and unfamiliar nature of RT can affect the patient’s experience of the treatment. Misconceptions or a lack of knowledge related to RT processes can increase levels of anxiety and enhance feelings of being unprepared at the beginning of the treatment. Moreover, the waiting time is often fairly long. Cancer care involves several, often independent clinics. Even if the clinical pathway is clearly described, transitions and information exchange can be problematic. RT is only provided at the university hospital in the region, with long distances and long waiting times for many patients.

The Digi-Do tool consists of two separate mobile apps: (1) an app providing a guided digital tour of the RT department, where the patient can familiarize themselves with the department by using virtual reality glasses; and (2) an app with additional information, including questions and answers, practical information, and short animated films about the RT process. The design of both apps was developed in a co-design process with patients and staff [43]. The primary aim of the researcher or innovator was to evaluate whether a digital information tool with virtual reality technology and preparatory information was able to reduce distress and enhance the self-efficacy and health literacy of patients with breast cancer before, during, and after RT. A secondary aim was to explore whether the digital information tool increased patient flow while maintaining or improving the quality of care [44].

#### Case 4: A Point-of-Care Dashboard for Schizophrenia Care (the PoC Dashboard)

The Department of Schizophrenia Spectrum Disorders at Sahlgrenska University Hospital delivers specialized care for people with psychotic disorders in the metropolitan Gothenburg area (with a population of approximately 600,000 people) in Sweden. Schizophrenia is the most common diagnosis among the approximately 3000 patients who receive care at the department’s 7 outpatient units. Approximately 20% of these patients also need acute inpatient care at 1 of the department’s 4 wards each year.

With the aim of supporting patient coproduction of health, a digital dashboard was developed to be jointly reviewed at the point of care by patients and case managers and psychiatrists to support evaluation and planning, outcome questionnaires, and patients’ care plans [45]. The dashboard was developed between 2016 and 2018 and was piloted at 2 outpatient units with approximately 400 patients for 18 months. The dashboard is one of several connected applications and displays for visualizing data fed by multiple systems to support, for example, planning, management, and triage and includes a unit-level overview of quality indicators to identify patients at risk. The dashboard project also served as a case in the development of the NASSS-CAT [20].

### Study Procedure

This study followed an iterative process, including analyses, discussions, and seminars (Figure 2).

As we used the NASSS-CAT, the analysis was deemed to be of an exploratory, deductive nature. First, an individual assessment of each case was made, and then the 4 cases were compared to find similarities. However, while using the NASSS-CAT in each of the 4 cases, the innovators had discussions about how to interpret the questions in the 7 domains. An additional method, namely, constant comparative analysis (CCA), was chosen as it is appropriate in collaborative projects to facilitate and identify agreements and disagreements [46,47] (Multimedia Appendix 2). After agreeing on how to interpret the questions, members of the IPF were invited to complete the analysis in a workshop.

In total, 4 bottom-up innovators had individually experienced complexities during their respective innovation processes from 2010 to 2019.

1. The 4 innovators got together and started the study in January 2020 by learning how to use the NASSS framework based on the work by Greenhalgh et al [15]. Support was available as one of the authors had been involved in the development of the NASSS-CAT [20].
2. The innovators identified complexities in their individual projects using the NASSS-CAT [20] in 2020.
3. During >30 one-hour meetings using the NASSS-CAT and taking minutes, the innovators identified, compared, and discussed similarities and differences regarding complexities in their respective innovations. A CCA was included in the process and is described in Multimedia Appendix 2.
4. A seminar was held in April 2020 with one of the bottom-up innovators and the IPF presenting the concept of complexity and the NASSS framework.
5. In October 2020, another seminar was held with the 4 innovators and staff members from the IPF to discuss the domains of the NASSS-CAT, illustrated by examples and findings from the assessment of the 4 bottom-up innovations. The participants from the IPF discussed and reflected on experiences of complexities. The seminar was recorded and summarized in a report in collaboration with a representative from the IPF [48].
6. The authors were commissioned to write a report (in Swedish) for the IPF exploring and summarizing the NASSS framework and complexity with examples from the bottom-up innovations [48].
7. The insights gained into the role of complexities from the entire aforementioned process were discussed and summarized and are presented in this paper.

## Results

In this retrospective exploration of the role of complexity in 4 bottom-up health care innovations in a Swedish region, both similarities and differences emerged among the 4 cases when using the NASSS-CAT (Multimedia Appendix 3). The findings for each domain are described in the following sections.

### Complexity Domain 1: The Illness or Condition

This domain has no or low complexity when the illness is well known and an assessment can result in a well-defined diagnosis and when there is, furthermore, knowledge and know-how regarding how to treat the condition successfully. Complexity can be related to conditions with less known causes, a high prevalence of multimorbidity, and challenging sociocultural factors (eg, language barriers). In our study, the cases that addressed mental illness (PoC Dashboard and MoodMapper) and diabetes (D-Foot) had more complexity related to the actual illnesses than the case aiming to prepare women before RT for breast cancer (Digi-Do) [49,50]. Both bipolar disorder and schizophrenia are strongly connected with comorbidity and lifestyle-related conditions, and even though national guidelines exist, there is no simple pathway to treat those conditions. The third case (D-Foot) involved diabetes, also an illness defined as complex due to the patient being treated in various institutions and with several lifestyle factors influencing the outcome of the treatment [39].

### Complexity Domain 2: The Technology (or Other Innovations)

An innovation is less complex if it is well known, ready, and easy to use and has a clear supply model, well-defined ownership regarding its intellectual properties, and low or no dependency on other systems. For the actual technologies under study, similarities regarding complexities revolved around interdependencies with other IT systems, ranging from local to regional and even national systems. Even if the technology already existed (D-Foot) or if new software was developed to create a better overview of data in several existing systems (PoC Dashboard), it was difficult to develop the innovation so that it enabled adoption beyond the local settings. Regulations regarding software used as a medical device [30] sometimes prevailed over the simple adaptations for different target groups. “Fireproof” walls exist between organizations (eg, municipal care, primary care, and specialist care), and different versions of the regional information systems, being related to ownership, budget, and management, make it less clear whether and how new technologies can be bought, adapted, and used in a local setting. In contrast, the Digi-Do app does not require any interaction with existing IT systems and was not deemed to be a medical technical device. The MoodMapper app, on the other hand, is complex as it aims to interact with both patients and health care staff, requiring interaction with medical electronic health records as well as ensuring a very high level of security to safeguard the patients’ integrity [51]. The need for supply chains included both purchasing and procurement and clinical implementation, the latter involving questions regarding intellectual properties (is the owner the bottom-up innovator or is it the region?), ownership management (which regulation steers the region when managing a medical device owned by the region?), and updates and maintenance of the eHealth tools (which department in the VGR is responsible for updates and maintenance of the innovations?). All the cases had run into or expected to run into severe complexity when planning to launch their innovations. It was clear that complexity regarding supply chains had not been considered by either the innovator or the VGR when intending to expand from a local level to a regional or national one. An example of the complex challenges related to the spread and maintenance of one of the eHealth tools, the D-Foot, is presented in the following paragraphs.

Regulations regarding funding and ownership made it difficult to implement a supply chain outside the local region as each of Sweden’s 21 regions has its own procurement processes. Since the start, the D-Foot project had been aiming for national spread. In 2017, the IPF approved funding with the aim of testing, Conformité Européenne (CE) marking, and thereafter implementing the D-Foot first in the VGR at the department of prosthetics and orthotics and then nationally. At the same time, several departments of prosthetics and orthotics in other regions were interested in using the D-Foot as soon as the CE marking was finalized. However, in June 2017, an official at the VGR decided that the region was only able to allow the D-Foot to be used within the region (Article 5.5 in the Regulation [European Union] 2017/745) [30]. Following this decision, national spread was impossible. The bottom-up innovator continued to have a dialogue with the IPF seeking a solution for national spread. In 2020, an opportunity for national spread arose by registering the D-Foot as a national medical information system (NMI) at the Medical Products Agency. An NMI is an information system developed for joint use at nationwide, regional, or municipal level in Sweden.

Thus, the D-Foot transitioned from being a “self-manufactured medical device” to becoming an NMI registered with the Swedish Medical Products Agency in 2020. However, the NMI registration was withdrawn by the VGR in 2021 due to new regulations from the Swedish Medical Products Agency [52]. The D-Foot remains a separate software program not integrated into the standard medical record system in the VGR. As a result, one option remained for national spread, namely, to CE mark the D-Foot, a procedure that was not as yet allowed or tested in the region.

### Complexity Domain 3: The Value Proposition (Costs and Benefits of the Technology)

Complexity in this domain arises when determining the value provided by the innovation to developers, users (patients, staff, and health care systems), and the broader health care ecosystem. Despite their origin as bottom-up innovations aimed at improving care, the complexities of demonstrating supply-side value in terms of business models and monetary benefits were challenging. Key questions included defining improved value, decision-making processes, the inclusion of nonmonetary values, and the extent of evaluation required: what is regarded as improved value? Who decides? Is it only monetary or other types of value as well? How much does the innovation need to be evaluated and how?

The Digi-Do has a defined regional vision and has faced challenges in quantifying value, especially regarding soft values such as reduced distress and increased health literacy and self-efficacy. The Digi-Do aims to optimize the use of waiting time before RT, adding value by reducing waiting times and queues. Furthermore, the innovation aims to create value for patients by delivering information in a novel, accessible format, potentially improving health literacy even for those with language difficulties or cognitive impairments. It also extends benefits to the patient’s social network, enhancing support and knowledge and reducing distress among family and friends affected by the patient’s cancer diagnosis. By using the often idle waiting time for meaningful preparation, the innovation may foster a sense of control and inclusion, diminishing distress and worry. Well-prepared patients may navigate the system more efficiently, potentially reducing waiting times for information dissemination.

The evaluation of the outcomes in this specific project is still ongoing through an unpublished randomized controlled trial [44], but so far, the qualitative results show a high level of acceptance of and positivity toward the tool. Nevertheless, there needs to be a discussion about how to endorse a more pragmatic evaluation of both effectiveness and process outcomes [53].

If successful, this approach could be adapted for other health care domains, although commercialization is not the project’s primary goal. Measuring soft values has proved challenging as they might not directly impact traditional health care outcomes. The other cases faced similar difficulties in pinpointing the exact stages in which costs and values could be calculated.

Enhancing foot health can improve the quality of life of patients and reduce health care costs associated with treating DFUs and amputations. Objective risk assessment by using the D-Foot precedes interventions, aligning with the vision of providing equal, high-quality care to citizens. Early interventions in the prevention process (D-Foot) might require more resources within primary care but were expected to be cost-effective in the long term due to a reduction in specialist care following fewer ulcer treatments and amputations [31,42]. In terms of quality of life and cost reduction, the value proposition needs further evaluation over a longer period relying on data related to care costs for at-risk patient groups. The D-Foot database contains valuable information on risk groups and foot status, serving as a data source for audits and evaluations to optimize foot care. It could also function as a quality registry, potentially becoming the new diabetic foot register in Sweden.

The MoodMapper aims to provide a more objective risk analysis and early interventions, potentially preventing hospitalization. In the examples of innovations (MoodMapper and PoC Dashboard) designed to prevent relapses in severe mental illness by coordinating data or even by asking patients to send and react to data, the need for hospital care could be reduced. However, this area is as yet unexplored.

The value proposition of the PoC Dashboard remains uncertain. Case managers and patients find the technology useful based on preliminary data. Local testing and piloting suggest perceived effectiveness, although the degree of cost-effectiveness is still unknown. The dashboard streamlines administrative tasks for staff, offering an overview of patients’ progress and risks while facilitating collaborative care planning. However, the technology’s potential as a commercial product is uncertain, mainly because it is integrated with older systems. Additional uncertainties involve the IT department’s role in dashboard maintenance and associated costs.

### Complexity Domain 4: The Intended Adopters of the Innovation and Technology

Complexity in this domain is higher when adopting the innovation, necessitating changes in routines, roles, and identities. Innovations that support existing routines with minimal disruption are associated with lower complexity, and all 4 cases required either behavior changes by patients or modifications to work routines for health care staff. For example, the PoC Dashboard simplified patient overview and reduced administrative work for staff, thus positively impacting daily tasks [54].

However, transferring the D-Foot to primary care posed challenges as different health care professionals (podiatrists, nurses, and physicians) with varying roles and routines questioned its added value. The MoodMapper required patients to trust the handling of their behavioral data, which could be challenging for those with symptoms of paranoia.

### Complexity Domain 5: The Organization Implementing the Technology

Complexity in this domain pertains to the efforts required to plan, implement, and monitor the innovation’s adoption, as well as to the organization’s overall capacity for innovation. Challenges included a lack of clear pathways for support, making it necessary to find the right individuals at the right levels for consultations. Different organizational levels faced varying complexities, and despite a desire for innovation, built-in regulations sometimes hindered dissemination. For instance, regulatory obstacles prevented the national spread of the D-Foot.

### Complexity Domain 6: The External Context for Innovation

Complexity in this domain is influenced by the political, sociotechnical, and regulatory context, as well as by stakeholder groups and interorganizational networking. In Sweden, despite a national Vision for eHealth by 2025 [55], the existence of 21 independent regions creates complexity in decision-making for local, regional, and national development and for the implementation of digital health care innovations. For instance, there is a national initiative from the government to improve cancer care, but the regions are self-governed in terms of budget and implementation. This means that, even if the regional cancer center had a national assignment to improve cancer care generally and the RT process in the local region specifically, it has no mandate to implement the Digi-Do without the approval of the RT department at each separate regional hospital.

Furthermore, if an innovation needs to be integrated with the IT systems, such as in the other 3 cases, national initiatives can be ruled out by regional procurement, management supply chains, and European regulations regarding medical devices [30]. If regional support and the management of supply chains only permit regional use, there will be no dissemination, and thus, bottom-up innovations risk becoming only local or, at worst, experiencing the “death of innovations” after the initial project phase.

### Complexity Domain 7: Emergence Over Time

Complexities were identified in all 4 cases (Multimedia Appendix 3). When summarizing the complexities from domains 1 to 6, all the authors concluded that the complexities were likely to increase in the coming 3 to 5 years, probably due to advances in technology, unexpected events such as pandemics, international conflicts, and new regulations and standards. In the coming years, a new regional medical record system, Millennium, is planned to be implemented. For small bottom-up innovators, it is not yet clear how the implementation of Millennium will affect their innovations [37].

## Discussion

### Principal Findings

#### Overview

In this study, we conducted a retrospective, deductive, and exploratory analysis of 4 cases using the NASSS-CAT LONG. We intended to explore whether there were shared or individual challenges related to bottom-up innovation projects in the same health care region. The analysis itself was complex, but it resulted in both common and individual learning, and all but one case have moved forward, partly due to new insights gained that have made progress possible. By applying the NASSS-CAT in various projects, the authors learned several lessons, the most important of which are described in the following sections. After that, we discuss and reflect on the methodology and the need or suggestions for further research. Finally, we briefly present how the cases have developed since the analysis.

#### The Innovation Versus the System

As proposed by Rogers [18], the properties of the innovation affect its probability of diffusion within and beyond the organization. Through this study, we have become aware of the need to understand the “system” in which we are working to develop and adopt innovations and make effective use of those innovations. The NASSS framework sheds light on various perspectives that can either facilitate or hinder the adoption, scale-up, and spread of technological innovations. Before our projects, none of us had fully considered all these perspectives. During this study, complexity was found and highlighted, involving many issues related to the organization or system rather than the specific innovation itself. Multiple regulations must be considered, and regional procurement [56], management of supply chains, and European regulations regarding medical devices [30] can hinder the spread of innovation. A lack of necessary interorganizational networking further complicates matters.

#### Linear Logic Versus Dynamic Complex Processes

By applying the NASSS framework, we discovered how the innovation process and the training we had all had in evidence-based medicine and the research process were geared toward a linear process rather than embracing complexity. We also discovered that the complexity was mostly found outside the actual innovation and related more to the system that the innovation was supposed to live in and to regulations and legislation. More specifically, it related to the organization’s willingness to integrate new innovations and to questions regarding how to manage, maintain, and finance innovations.

Developing and deploying new bottom-up innovations in health care involves multiple logics [57]. Initially, we attempted to approach this in a traditional linear fashion with sequential steps from idea to widespread adoption. However, we quickly realized that this linear approach did not align with the reality of navigating the complexities of health care innovation. Instead of a straightforward innovation journey, it often felt like traversing a dense jungle, making it challenging, if not impossible, to gain a comprehensive overview of the landscape, identify opportunities, and predict the appropriate course of action.

Complex environments often require creative and dynamic thinking; in contrast, a linear approach may stifle the ability to respond to unexpected challenges or opportunities. Innovation is inherently uncertain and unpredictable [58]. It often involves trial and error, experimentation, and the willingness to explore unconventional ideas. A rigid stepwise approach may not accommodate the iterative and nonlinear nature of the innovation process. Complex organizations involve numerous interconnected variables and dependencies. A linear approach may overlook these interconnections, leading to suboptimal solutions or unintended consequences. Innovation often requires a holistic understanding of the organization’s ecosystem. This understanding is hindered if established cultures in the complex organizations are resistant to change [57]. A nonlinear approach may face resistance from employees or departments unwilling to deviate from established norms.

Successful innovation requires addressing cultural and organizational barriers, which may not fit neatly into a linear plan. Finally, complex organizations require adaptive leadership that can navigate ambiguity and inspire a culture of continuous improvement [59]. These are important findings as innovation, particularly in the realm of new technologies, is often seen as a potential solution to address the challenges facing health care. Calls for innovation and new ways of working have come from various sources, including governments, health care organizations, and life sciences clusters. However, the high failure rate of health care technology projects suggests that there may be deficiencies in the structure, resources, and knowledge needed for success [60]. Furthermore, there is a risk of simplifying the complex innovation process by building a support system that is linear. The linear and stepwise approach (first do this, then do that) is counterproductive. While a linear approach may work in certain situations, the nature of innovation in complex organizations demands a more flexible, adaptive, and nonlinear methodology. Embracing uncertainty, fostering creativity, and adapting to change are critical elements that a rigid stepwise approach may not adequately address in the context of complex organizational innovation [58].

#### Value

For all 4 cases, questions arose related to value and costs. Will there be an initial or a recurrent cost for the product, or will the cost be related to a new service that entails new tasks for staff? There is an advantage in specifying both costs and values, as well as the effect of the innovation on other resources, early in the innovation process. Therefore, health-economy analyses are needed, but they are difficult to design and perform as some innovations focus on increasing soft values that are difficult to translate into monetary variables.

Indeed, evaluating the values—different kinds of values and on different levels—of health care innovation is complex. While clinical testing can demonstrate its usefulness to end users, it is often difficult to determine whether the outcomes involve soft values (eg, reduced distress and improved health literacy and self-efficacy) or hard, monetary values [14]. Furthermore, the distribution of costs and value resulting from an innovation can be intricate, making it hard to assess. Questions arise about the initial and recurrent costs and whether they relate to the product or to new services that require additional staff tasks. Early in the innovation process, there is a need to specify both costs and values, be they monetary or qualitative. Clearly describing and anchoring a value proposition, whether it involves soft or hard values, with stakeholders early in the process is crucial for understanding costs and outcomes. However, finding effective ways to evaluate an innovation before it is ready for large-scale testing can be challenging. Similarly, value and costs stemming from an innovation can be distributed across the organization or organizations in ways that are difficult to assess. Calculating the health costs of improving care processes that involve many actors in a complex organization such as the VGR is complicated [61]. More pragmatic evaluations of both effectiveness and process outcomes are needed and can help show the effect from different angles [53].

For all cases dealt with in this study, the value proposition in terms of quality of life and cost reduction needs further evaluation over a longer period relying on data related to care costs for at-risk patient groups. The D-Foot database contains valuable information on risk groups and foot status, serving as a data source for audits and evaluations to optimize foot care. It could also function as a quality registry, a new diabetic foot register in Sweden. In the MoodMapper, the users comprise patients; their clinical teams; and, occasionally, relatives or caregivers. A published study highlights the value of implementing and receiving psychological relapse prevention for these groups, leading to improved understanding of bipolar disorder [62] that might, in turn, lead to enhanced working relationships and better condition management. However, the evidence is not consistent, and further studies are needed [61]. Moreover, for patients with bipolar disorders, having some of their behavioral patterns (such as step count and estimated sleep) automatically monitored meant that there needed to be a great deal of trust in how data are handled, something that might be difficult for patients experiencing symptoms of paranoia.

#### Co-Design and Coproduction

Involving users both directly and indirectly at an early stage of the development process is highly beneficial, particularly because what benefits one person may pose challenges for another, thereby creating complexity. Although there are several examples of how coproduction is useful in the innovation process, the existence of complexity must not be neglected in the co-design.

Bottom-up innovations in care encompass a wide spectrum of patient-centric approaches, empowering individuals and communities to actively participate in projects aiming to support well-being. These innovations, driven by the challenges that health care faces, range from self-management tools [62-64] and patient support networks to community-driven health programs [6-8,10,59-61,65-67]. They appear with different approaches, such as lean production [9] and Six Sigma [68]. Coproduction can enhance the 3 Rs in research—reach, rigor, and relevance [69]—by ensuring that the right needs are addressed and that the innovation is practical for both patients and staff.

Enthusiastic innovators and staff should be engaged early in the process, along with representatives from patient organizations or individuals with relevant experience. There is a strong movement toward involving patients in health care improvement, and genuine engagement is necessary for truly bottom-up innovation involvement [70] as it can lead to more radical solutions or suggestions when used correctly [71]. If a technological innovation is too demanding or unfamiliar for users, it is unlikely to be accepted. Piloting with stakeholders is crucial for assessing practicality [59], and using input from stakeholders in the right phase can increase the possibility of finding radical suggestions, as well as saving time for both parties (developers and patients) [71]. We support the idea that coproduction incorporating the multifaceted aspects of complexity is necessary in the evaluation of success in the implementation of bottom-up innovations [4].

#### Methodological Considerations

Performing a retrospective, deductive analysis as a case study [21] with 4 cases with differences regarding where they were in the innovation process and with different technical solutions was challenging, but it provided multiple valuable insights. The authors found that, before using the NASSS-CAT, users need to be familiar with the NASSS framework [15]. The NASSS-CAT appeared deceptively easy at first, but it was more difficult to use and more time-consuming than expected. A need for a way to track how we could jointly understand and agree on the meaning of the NASSS-CAT by using CCA became apparent during the work, leading to a common language being agreed upon and a consensus being reached on how to interpret the terminology used in the NASSS-CAT. During the CCA, discussions about how to interpret the questions in the 7 domains of the NASSS-CAT took place in cycles, and thus, it was a continuous learning process. As intended by the method [46,47], finding disagreements and negotiating led to a higher degree of understanding not just of the instrument but also of the concept of complexity. The 4 innovators contributed multiple perspectives based on their own cases and discussed their different understandings of the narratives, the domain questions, and the subquestions. As the authors used a nontranslated version of the NASSS-CAT and are native speakers of Swedish and not English, the CCA helped them understand the questions in the NASSS-CAT. Therefore, the use of CCA statements and negotiations on how to interpret the questions in the NASSS-CAT facilitated the analysis and helped create a common language within the group.

The NASSS framework was developed through a detailed review of the existing literature and clinical cases [15,20], but to our knowledge, the tools (NASSS-CAT) have so far been sparsely tested for their ability to unveil complexity in bottom-up projects in public health care. Going from commonly used methods for quality improvement (eg, using the Plan-Do-Study-Act method [72] to incorporate complexity assessment) shows promising results. A recent study used the framework and tool combined with the Plan-Do-Study-Act cycles of improvement to plan and evaluate digital services for patients in Sweden [73]. Similar to our retrospective analysis, that study identified several elements of complexity, explaining a gap among the capacity of adopters, the organization, the wider system, and how intended users valued the service. This gap hindered the innovations from integrating new services into routine care effectively [73]. Similarly to us, these authors found the tool and framework helpful in that they allowed for deeper insights into the project compared to only following method, approach, or cycles or other tools or models for innovation. It seems that, even if complexity is revealed early in the process, this still does not solve the problems. However, if people working with innovation or in supporting innovation become more aware of complex elements, issues might be easier to anticipate or even deal with earlier. Such awareness can thereby help explain obstacles and prevent failure, hence enabling more successful innovation projects in health care, as presented by Greenhalgh et al [15].

#### Strengths and Limitations

The strength of this study lies in the 4 different cases representing both somatic and psychiatric care and the innovators’ long experience in both health care and eHealth. The diversity of innovations presented and the different departments that each of the innovators worked in contribute to a broad overview of shared experiences. None of the innovators had worked together before this study. The fact that all cases came from the same region with the same support function strengthens our results by showing that (1) knowledge of complexity needs to be improved in such systems and (2) the project itself contains complexity in different domains even if we found several common problems. Therefore, the study increased the understanding of the role of complexity, not only in the studied bottom-up innovations but also in the system in which the innovations took place, through prolonged engagement [50]. This study was strengthened by the support of one of the authors, who was involved in the development of the NASSS-CAT [20], but despite this, it appears that adaptation to the setting (geographic and cultural) is crucial.

The retrospective NASSS-CAT analysis of 3 of the 4 cases was mainly performed by the respective innovators without direct input from stakeholders involved in each of the cases. This meant that only 1 perspective from the many actors involved in each of the projects was put forward. The rationale for this was that each innovator had already faced and, therefore, was acquainted with the diverse complexities addressed in all 7 domains. However, other perspectives might have further improved the analysis. The PoC Dashboard project was assessed regarding complexity in a workshop with stakeholders and discussed with management [54].

Even though the NASSS-CAT tools have been used previously [74], more testing in clinical bottom-up innovation cases is needed to scrutinize their utility in a Swedish setting and to learn from the experiences originating from 4 different cases that used the tools.

#### Use and Usefulness of the NASSS-CAT

In this section, experiences of the use and utility of the NASSS-CAT are presented. At the start, the 4 bottom-up innovators were naïve and expected the NASSS-CAT [20] to be easy to comprehend and use as they identified complexities in their own innovations. As mentioned previously, by using the CCA interpretations from multiple perspectives (the 4 cases), a shared understanding and language regarding how to interpret the questions in the NASSS-CAT was established. The results from the CCA revealed that each of the 4 innovators needed to clarify or consider a number of points in their own NASSS-CAT analysis while assessing complexities in each of the domains. The most important issues were as follows:

1. To define the time frame and the scope that the innovator is assessing.
2. To define the intended users and adopters at the time of the studied project.
3. To rethink the way in which the value proposition can be measured.
4. To consider that questions in the NASSS-CAT regarding ownership; supply chains; and use and spread at the local, regional, national, and international level belong to both “Domain 5: organization” and “Domain 2: technology.”

#### Moving Forward in Supporting Bottom-Up Innovation

This study explored insights from the NASSS framework, revealing that the adoption and dissemination of technological innovations are influenced by organizational and systemic factors rather than by the innovations themselves. The success of bottom-up innovators in navigating complexities emphasizes common challenges across innovations. The NASSS framework has illuminated various perspectives that can either facilitate or impede the adoption, scale-up, and dissemination of technological innovations.

The 4 bottom-up innovators managed to navigate through the complexities within the innovative system, uncovering overarching challenges that unified their respective innovations. However, it is essential to recognize that the NASSS-CAT cannot be used as a linear checklist. Existing support systems, while aiming to foster innovation, may unintentionally follow a linear approach rather than embracing frameworks suitable for complex interventions, such as the Medical Research Council guidance [75]. To better support health care innovators, a “midway filter system” is needed, which offers profound insights into innovation within complex systems. Implementing such a filter between top-down and bottom-up approaches would facilitate bidirectional knowledge transfer. It would enable clinical insights, ideas, and innovations to be discussed in harmony with the regulatory framework, ultimately leading to improved and equitable health care as envisioned by Tierney et al [10], who found that localized, regional, and flexible innovations can shape care in the future [10]. Incentives to connect bottom-up initiatives with a top-down vision at a national level in building systems for digital innovation and health IT are presented by Sheik et al [67] from the United Kingdom. We share their vision to improve usability and interoperability and integrate bottom-up with top-down resources.

Our study, similarly to the research by Batalden and Davidoff [76], discusses the complexities of integrating grassroots idea innovations into established health care systems. Batalden and Davidoff [76] highlight the need for organizational changes and a shift in culture to recognize the value of patient-driven innovations and effectively incorporate them into clinical practice.

Future studies should consider a translation project of the NASSS framework from English into Swedish. This would facilitate the framework’s use in a Swedish context, similar to the translations of health-related quality of life questionnaires, which follow guidelines to ensure validity in terms of language and culture [53]. In addition, an evaluation is recommended alongside updates to the NASSS-CAT. Some subquestions may benefit from further splitting, such as assessing the likelihood of technology obsolescence or the measurement of alternative ways to evaluate innovation. It is crucial to involve relevant stakeholders in these changes. Cultural adaptation should receive significant emphasis to provide a language that is relevant to the Swedish context. We also suggest that future studies explore similarities and differences regarding the existence of complexities when bottom-up innovations are developed and implemented in other regions. Finally, we consider making a follow-up prospective evaluation of our 4 innovations. By doing this, we can possibly review the impact of this study on the long-term outcomes of each innovation using the NASSS-CAT LONG.

### The Progress of the 4 Cases

The insights gained from our exploration of the existence of complexities in innovation processes led to some of the presented innovations being appreciated in the VGR. The Digi-Do and the D-Foot have gradually, during the study, been acknowledged as important in building future care with digital tools. The VGR has granted the innovator of the D-Foot the legal rights to be spread nationally and internationally and to be implemented and scaled up to prevent DFUs through early screening.

The Digi-Do has been evaluated, and the results show a positive effect on the users, indicating reduced levels of distress and an improved sense of preparedness [43,77]. Hence, the difficulties in evaluating soft values have been successfully dealt with. Since the analysis presented in this paper, the intellectual properties have been transferred to the VGR together with the RT department, and updated versions of the Digi-Do are underway.

Learnings from the complexity assessment of the PoC Dashboard [54] helped address the challenges differently by going for a simpler technical solution with less dependencies on other information systems and focusing on core features such as supporting patients and health care professionals in the planning and evaluation of care. This was done by adapting Dialog+, which is both a tool to measure and monitor patient-reported outcome measures and patient-reported experience measures and a solutions-focused methodology, to fit Swedish psychiatric care [78]. It has since then been piloted and tested for >4 different patient groups in mental health care settings and is being implemented as part of routine psychosis care. The MoodMapper is not an active innovation project in Sweden. However, it is used internationally in research to map behavior changes in mental disorders [79,80].

### Conclusions

The NASSS framework increased the bottom-up innovators’ understanding of the role of complexity in their innovations. The analysis provided valuable insights by identifying and bringing attention to complexities, particularly within the broader system, albeit requiring a deep understanding. This study enriched our comprehension of the pervasive role of complexity in bottom-up innovations within public health care and shed light on the practical utility of the NASSS-CAT. Early use of a validated tool aids in identifying complexities and pinpointing the domains in which these complexities exist. Importantly, even when bottom-up innovations arise within the same support organization, the complexity can vary based on the developmental phase and the unique characteristics of each project. Identifying, defining, and understanding complexity may not solve the issues but substantially improves the prospects for successful innovation implementation provided the right expertise is available to support the process.

Successful innovation within complex organizational structures necessitates a comprehensive understanding and an adaptive leadership to surmount cultural resistance and organizational impediments. A rigid, linear, and stepwise approach risks disregarding interconnected variables and dependencies, leading to suboptimal outcomes. Success lies in embracing the complexity with its uncertainty, nurturing creativity, and adopting a nonlinear methodology that accommodates the iterative nature of innovation processes within complex organizations.

## Appendix

Multimedia Appendix 1 Description of each of the 4 cases of bottom-up innovations in Swedish health care, including a presentation of how the innovations relate to the 7 domains of the nonadoption, abandonment, scale-up, spread, and sustainability complexity assessment tool.

Multimedia Appendix 2 A constant comparative analysis of how to interpret the 7 domains of the nonadoption, abandonment, scale-up, spread, and sustainability complexity assessment tool.

Multimedia Appendix 3 Complexities in the 4 bottom-up innovations assessed using the nonadoption, abandonment, scale-up, spread, and sustainability complexity assessment tool.

## Abbreviations

CCA: constant comparative analysis
CE: Conformité Européenne
DFU: diabetic foot ulcer
IPF: Innovation Platform
NASSS: nonadoption, abandonment, scale-up, spread, and sustainability
NASSS-CAT: nonadoption, abandonment, scale-up, spread, and sustainability complexity assessment tool
NMI: national medical information system
RT: radiation therapy
VGR: Region Västra Götaland

## Data Availability

The data sets generated during and analyzed during this study are available from the corresponding author on reasonable request.
