# Supplementary material for: Exploring the Role of Complexity in Health Care Technology Bottom-Up Innovations: Multiple-Case Study Using the Nonadoption, Abandonment, Scale-Up, Spread, and Sustainability Complexity Assessment Tool
Source: JMIR Hum Factors. 2024 Apr 26;11:e50889. doi: 10.2196/50889 (PMC11087855; doi:10.2196/50889)
Supplement: Multimedia Appendix 1 [file humanfactors_v11i1e50889_app1.docx]

**Table S1.** Description of each of the four cases of bottom-up innovations in Swedish healthcare, including a presentation of how the innovations relate to the seven domains in the NASSS-CAT framework [20].

| **Categories & Narratives** | **Digi-Do** | **MoodMapper** | **D-Foot** | **Point-of-care dashboard (PoC)** |
| --- | --- | --- | --- | --- |
| State of project during analysis: Planning/Ongoing/Evaluation | Evaluation | Ongoing | Ongoing | Ongoing |
| Geographic area: Local/Regional/National | Regional | Local | National | Local |
| Users/ conditions | Patients diagnosed with breast cancer about to commence radiotherapy treatment (RT). | Adults diagnosed with bipolar disorder registered within a specialist public mental health service. | Patients diagnosed with diabetes visiting a department of prosthetics and orthotics (DPO). | Adults diagnosed with psychosis registered with a specialist public mental health service. |
| Technology | A digital information tool consisting of two coherent apps, providing 1) a digital visit to the RT clinic using VR technology, and 2) practical information with links, Q&As, and short animated films. | Mobile phone application with background monitoring of daily activity patterns via built-in sensors in the users’ phone. | A software application functioning as a decision support system, accessible for certified prosthetists and orthotists (CPOs) on the intranet in Region Västra Götaland (VGR). | Point-of-care dashboard offering a visual overview of progress (including questionnaires for symptoms and functioning) and planning. |
| Level of care | High specialist care | Secondary care | Secondary care | Secondary care |
| Main goal of the project | Prepare patients better before RT by using the waiting time; less distress and faster patient flow. | Identifying early signs of a possible relapse so that preventive measures can be taken. | Identifying patients at risk of developing diabetic foot ulcers (DFUs) and, based on that risk, patients are treated according to the national guidelines [25, 26]. | Improving patient engagement with their own care; care planning; audit. |
| Policy context | Increasing demand for RT, implying long waiting times; patients with a high level of distress due to cancer diagnosis and unknown high-tech environment. | High cost of bipolar disorder driven by relapses and hospitalisations. | Preventive care of feet has been low prioritised, leading to DFUs and amputation. | Large numbers of patients diagnosed with schizophrenia, who are seen as a ‘vulnerable group’, with low engagement and compliance. |
| Project framed primarily as | Using meaningless waiting time for meaningful preparation. | Prevention of relapse in serious mental illness. | Prevention of DFUs. | Empowering patients. |
| Project status (+test/volume/research) | Action research project. Version 1.0 tested as a pilot project with 15 patients. Version 2.0 developed to be tested in a randomised controlled trial with 160 patients (80+80). | Implementation. Results from seven patients over four months subject to AI trial analysis. | Implementation. Tested for > 9 years, at four DPOs in the VGR (n > 250 patients; n =13 CPOs)[27, 28]. | Local quality improvement project. Tested for > 2 years at two outpatient units with > 400 patients. |
| Key participants | Clinicians, patients, researchers, app developers. | Patients and their family, clinicians, researchers, patient representatives. | Clinicians, patient representatives, managers, statisticians, researchers, IT programmers, support staff. | Clinicians, managers, IT support staff. |
| **1. THE CONDITION OR ILLNESS** | Breast cancer is the most common cancer diagnosis for women, with approximately 9,000 new cases/year in Sweden. The survival rate is 86%. There are national guidelines for treatment and standardised care plans to speed up the process. Approximately 75–80% of patients will commence RT as a complementary treatment. Long waiting times, combined with long travel distances, are common, and normal treatment time is three weeks, five days/week. | Bipolar disorder is a mental condition typically diagnosed in early adulthood, often requiring lifelong treatment. It causes abnormal shifts in mood, activity levels, and the ability to carry out day-to-day tasks. Moods range from periods of being extremely ‘up’ (known as manic episodes) to being very ‘down’ (known as depressive episodes), lasting for several days or weeks. Even with proper treatment, mood changes can still occur. Treatment is more effective when a patient and healthcare provider work together and talk about concerns and choices. | The lifetime risk of developing DFUs is estimated at up to 34% [23]. Effective prevention strategies can reduce this risk by half, improving individuals' quality of life [24]. There was a need to develop software for early screening of risk factors that could generate an objective risk stratification based on multiple assessments and aligning with current guidelines [25, 26]. | Schizophrenia, recognised for its complexity arising from high multimorbidity, demands multimodal treatment and coordinated care across various health and social care providers. Despite considerable research on schizophrenia and multimodal treatment effectiveness, challenges persist in coordinating multiple providers. Cognitive impairment in those diagnosed with schizophrenia, influences insight and motivation, impacting treatment adherence, and potentially leading to involuntary care and coercive measures. Access to personalised treatment, housing, and support, shows significant variability. |
| **2. THE INNOVATION/ TECHNOLOGY** | The innovation has been developed together with staff and patients and tested as a pilot project. The results show great interest from users, but some amendments have been made to version 2.0. The Digi-Do is a digital information tool consisting of two coherent apps, providing 1) a digital visit to the RT clinic using VR technology, and 2) practical information with links, Q&As, and short animated films. The patient obtains access to the tool post-surgery when informed about RT, in order to prepare at home by becoming familiar with the RT environment and obtaining information when it is most suitable. | Mobile phone application with background monitoring of daily activity patterns via built-in sensors in the users’ phone. Programmed by a researcher and, during this phase, pilot tested. | Version 1.0 of the D-Foot software was developed from 2011 to 2016 by an expert group comprising CPOs, patient representatives, and orthopedic surgeons in the VGR [27]. Initially, it underwent regional testing with positive results. Based on the users’ comments, improvements of the software have been made [28]. It is a ‘self-manufactured medical device’ [30] not integrated into the standard medical record system in the VGR. | The dashboard was developed within the department in collaboration with other psychiatric departments at the hospital. It has significant technical interdependencies with systems controlled by the regional IT department. The development of the dashboard has been intertwined with older systems, making use of work processes already in place. There is uncertainty about how to adapt the technology to enable scale-up across the whole department. The extent to which the technology will be obsolete within three to five years is unknown, but the IT department is planning the broad implementation of other new health information systems within that timeframe. |
| **3. THE VALUE PROPOSITION** | Value will be created for patients by providing information in a new, accessible way. Health literacy can be positively affected, even for vulnerable groups with language difficulties or cognitive impairments. By also allowing the social network around the patient to acquire an understanding of the upcoming procedure with RT, the innovation can provide greater support and knowledge; as a result, it can reduce the distress of family and friends who are often affected negatively by a loved one’s cancer diagnosis. By using the often, for the patient, meaningless waiting time for meaningful preparation, the innovation might enhance the feeling of being in control and included and thereby diminish distress and worry. A well-prepared patient might go through the system more rapidly, thus freeing up time to shorten queues by needing less time for information. | Recurrence rates for bipolar disorder are high, despite effective treatments with mood stabiliser drugs. Self-help treatments that teach patients to recognise and manage the early warning signs of impending manic or depressive episodes, are popular with patients. The main aim of these interventions is to intervene at an early stage and prevent bipolar episodes, thereby preventing hospitalisation. However, these signs can be diffuse and/or difficult to identify. As the disorder will affect daily functioning, the app can contribute to a more objective risk analysis and to early interventions. | Enhancing foot health can improve the quality of life for patients and reduce healthcare costs associated with treating DFUs and amputations [29,31]. Objective risk assessment precedes interventions, aligning with the vision of providing equal, high-quality care to citizens. In terms of quality of life and cost reduction, the value proposition needs further evaluation over a longer period, relying on data related to at-risk patient groups regarding care costs and environmental costs [32,33]. The D-Foot database contains valuable information on risk groups and foot status, serving as a data source for audits and evaluations to optimise foot care. | The value proposition of the project is uncertain. Case managers report finding the technology useful, as do patients, according to preliminary data. Local testing and piloting have generated evidence of perceived effectiveness, although the degree of cost effectiveness remains unknown. The staff spend less time on related administration. The dashboard provides an overview of patients’ progress and risks and supports the collaborative planning of care. The technology’s potential value as a commercial product is uncertain and probably impossible to assess, as the new technology is interwoven with older systems. Additional uncertainties are related to the IT department’s role in the maintenance of the dashboard and related costs. |
| **4. THE INTENDED ADOPTERS** | The primary users are patients, using the tool at home with no interaction with the healthcare staff through the tool. The secondary users are healthcare staff at the surgical clinic, who introduce the tool to the patient, and healthcare staff at the RT department, who meet the patient before and during treatment. At the current stage, no further administration will be needed, apart from the research team, who will provide the apps and VR glasses, but, if the tool is implemented, the administration needs to be incorporated in the organisation for planning and preparing for RT. | The intended service users are patients, their clinical team, and, in some cases, relatives/carers. According to a published study 34], the process of implementing and receiving psychological relapse prevention was valued by both service users and care co-ordinators for three similar sets of reasons: 1) improved understanding of bipolar disorder, 2) enhanced working relationships, and 3) improved ways of managing the condition. | Patients and healthcare staff, i.e., the CPOs, are the intended users. Administrative staff at the DPO might be involved if they book the patients. The VGR IT department is responsible for providing system maintenance. Researchers and statisticians are interested in the gathered meta data. | The domain of intended adopters is the least complex domain due to a perceived readiness within the organisation. The primary users are healthcare professionals in the care team, as well as patients during visits to the outpatient clinics. The secondary users include managers and administrators. The technology is expected to lessen the workload for administrators, as more tasks are completed at the point of care by the patient and healthcare professional. The dashboard pilot testing at two outpatient units for 12 and 20 months, respectively, indicated that the innovation is useful for both healthcare professionals and managers. Furthermore, participating healthcare professionals report that most patients use the dashboard with ease at yearly follow-up visits. Most patients would prefer their next visit to include the dashboard. |
| **5. THE ORGANISATION(S)** | The organisation for the improvement of cancer care – the Regional Cancer Centres (RCC) – is responsible for providing new and innovative ways of communicating with patients and also for improving RT processes. This has also been stated in a report on how to create an even better RT service in the region, where equal information was seen as being highly important. As a result, the project has been developed in co-creation with the departments and is wanted at the clinics. However, there are difficulties implementing IT solutions that have not been developed by the regional IT department and, because this project is a collaboration with external partners (Jönköping University), it has been impossible to obtain regional funding. Ownership after testing has been an issue for possible implementation and legal concerns have been difficult to address. Because there is no clear business case, other than providing better information, it has been difficult to find funding from med-tech grants. | Public healthcare services at a university hospital, where the care of bipolar patients takes place at a specialised outpatient clinic, as well as two inpatient units. The organisation is structured in a hierarchical way where the department has over 500 employees at nine inpatient units, seven outpatient clinics, and the largest psychiatric emergency room in Sweden. | The DPO at Sahlgrenska University Hospital is the largest DPO in Sweden with over 70 employees. In 2010, the DPO had little interest in digital solutions. Since 2018, the leaders have had a growing interest in the D-Foot, supported by visions from the VGR to go digital in healthcare [35,36]. | The department and the technology have a good organisation–innovation fit, as the innovation was developed in-house to support the organisation’s mission and ambitions. Digitalisation is perceived as a quality improvement strategy rather than a product development project. Horizon scanning has increased the awareness of innovative technologies, and the organisation has a tradition of supporting and trying new ways of working. In recent years, technological innovations have been a focal point, enabled by the recruitment of IT developers, and have been embedded in the organisation. It has been challenging to pilot and evaluate new technical innovations due to dependence on the regional IT department. The development of a dashboard is neither part of a regional initiative to develop the future health information system nor part of a product development plan with a clear business case. Internal support has made the pilot tests possible at the department, but the lack of sponsors at higher organisational levels, and uncertainty about the value proposition from a wider organisational perspective, add challenges within this domain. |
| **6. THE EXTERNAL CONTEXT** | The pending transition to a new health information system might affect the opportunity for implementation, but, because the tool does not provide interaction, this should be a minor risk. There are national and regional goals for digitalisation, which can both help and compete with the innovation, as it has the ambition to be scientifically evaluated before implementation. Other projects might catch up and use more modern technology. The project needs to expand to include more cancer diagnoses in order to be valuable for the staff at RT departments. | There is enthusiasm for innovations, but there is a strict process of quality and evidence control dictatating the methods that are implemented, in addition to there not being a specific budget for testing new methods. | At regional level:  The development and introduction of a new medical record system (Millennium) in the VGR is a challenge, because:   - small units such as DPOs are not involved in the workstreams that designed the system - the digital foot check by the D-Foot is not a highly prioritised software to be integrated in Millennium [37].   At national level:  Spread and implementation nationally are stopped due to the lack of a regional quality management system for medical devices [45] and due to a regional policy NOT to spread self-manufactured or CE-marked medical devices nationwide. The reason for this is that the region does not have the routines in place, as is described, in the EU regulations regarding medical devices [38]. | Changes in the wider context may impact the organisation and the introduction of the technology. In particular, the implementation of a new health information system can potentially crowd out efforts to deploy the dashboard technology within the department. There is an enormous drive for innovation and digitalisation in Swedish healthcare, in the form of either large national or regional projects that are deemed difficult to influence, or as small projects such as freestanding apps that are unable to make use of available healthcare data. There are few opportunities to learn from other organisations; almost no other organisations that use similar technologies exist, and if they do, they mostly concern patient groups other than those within mental health services. |
| **7. EMERGENCE OVER TIME** ^a^ | See Appendix 2, where the summary from the NASSS-CAT analyses is found. | See Appendix 2, where the summary from the NASSS-CAT analyses is found. | See Appendix 2, where the summary from the NASSS-CAT analyses is found. | See Appendix 2, where the summary from the NASSS-CAT analyses is found. |

Note: ^a^ In the summary domain, Domain 7, several complex aspects are considered that can change over time, e.g., rival technologies, policy priority, the organisation of the society [15]. In the result section, the innovators’ summary is found. See Appendix 2, where the summary from the NASSS-CAT analyses is found.
