# Supplementary material for: Exploring the Role of Complexity in Health Care Technology Bottom-Up Innovations: Multiple-Case Study Using the Nonadoption, Abandonment, Scale-Up, Spread, and Sustainability Complexity Assessment Tool
Source: JMIR Hum Factors. 2024 Apr 26;11:e50889. doi: 10.2196/50889 (PMC11087855; doi:10.2196/50889)
Supplement: Multimedia Appendix 2 [file humanfactors_v11i1e50889_app2.docx]

A constant comparative analysis of how to interpret the seven domains in the NASSS-CAT.

| **NASSS-CAT domain** | **Agreements** | **Disagreements** | **Negotiations** | **Action** |
| --- | --- | --- | --- | --- |
|  | Agreements that the group made regarding how to interpret the questions | What did we disagree about, interpret differently? | Conclusions that the group reached in order to complete equal/comparable analyses of our four innovations | How the negotiations affected the NASSS-CAT analyses of our own innovations |
| Narratives | - Short and coherent between examples | - What to include? - Write a narrative before or after answering complexity questions? - What is the scope? - Timeframe (start and stop) for analysis? | - Clearly define the scope: local, regional, or national - Decide the phase the project is in, e.g., evaluation or planning | - Put background example descriptions in another table or text, minimise text in narratives |
| **1. THE CONDITION OR ILLNESS** |  |  |  |  |
| There is significant uncertainty about the condition or illness | - Relatively easy to define actual illness | - Is not any illness complex for the affected person, or can an illness be generalised? - What if the innovation aims to address the potential difficulties, such as health literacy? - Is the condition affected by lifestyle-related factors or not? | - If most people with the illness had an uncomplex life before diagnosis, then it is not complex after diagnosis | - Keep the scope of analysis to the one initially decided |
| Many people with the condition have other co-existing illnesses or impairments that affect their ability to benefit from the technology or service |  | - How to define how many people are “many”? | - If an overrepresentation in society = many |  |
| **2. The technology** |  |  |  |  |
| There is significant uncertainty about what the technology is | - Yes, if: - the technology is under development - many ties have not yet been resolved - integration between systems is needed - uncertainty about whether innovation will be integrated into a new information system (as decided by politicians) - If not tested on a larger scale, the technical solution is not yet in place - Depends on the number of ties between actors, patients, innovation and care - Only made easier for information already existing (what do we mean?) | - From which perspective is the question answered? - Who is the user? - It can be easy for one user and very complex for another user - Difficult to maintain focus on individual scope and, as a result, difficulties with uncertainties that might appear in future perspectives - Is the innovation a completely new way of working or only something made more visible? - Are we uncertain whether the innovation will be affected by the new information system on the way towards being implemented (Millennium, as decided by politicians) | - Define and decide the perspective of analysis for each innovation | - Clearly define the main users of innovation |
| The technology appears to be ‘disruptive’ (i.e. it is likely to require major changes to organisational tasks and routines) |  |  |  | - Clearly define whether the innovation leads to a new way of working or just the same way but made easier |
| The technology (and/or the service model it supports) is likely to become obsolete or require replacing within the next three to five years |  |  | - Are we analysing the data collection/technology or the service model that is needed to support the technology? | - Split the last question into two separate ones |
| **3. The value proposition** |  |  |  |  |
| The commercial value of the technology is uncertain, e.g.:   - If the technology does not yet exist in a definitive form, the case for investing in its [further] technical development is weak. - The technology does not have a plausible business case, including up-front investment, a well-defined customer base and market drivers, consideration of competing products and a realistic assessment of challenges in implementing at scale in a public-sector health or care environment | - All innovations are tested in some way (pilot testing or on a large scale) - Each innovation was a better way of working compared with previous way of working - > increase the quality of healthcare - All innovations are related to public healthcare not aiming to yield profit | - What is regarded as improved value? - Who decides? - Only money or other values? - How much does it need to be evaluated and how? | - Value depends on the type of innovation - The innovations produce more gain than harm - RCTs are difficult or not necessary to perform to prove the value of the innovation - We can write not applicable in this domain | - Change answers to n/a - Specify how and whether evaluations are made according to the phase of innovation - Two different levels of evaluation (soft values for users and supply-side values) are needed. - Each innovation will define its own value - Read the narrative to this domain…what have you stated? Let the process be clear: see narrative – check each question and so on. |
| The value to the patient or client is uncertain | - More demands on evaluating the value for the patients rather than for the clinicians - Value difficult to differentiate between financial and other value (e.g., a broader value such as the sustainability effects of the innovation) |  | - New approach to value the proposition is needed |  |
| **4. The intended adopters** |  |  |  |  |
| There is uncertainty about whether and how patients and their carers will adopt the technology | - Always more difficult if more staff need to be involved…and with different professions - A lot of worries about new things from staff | - Depends on how tested the innovation is | - Clearly define the intended adopters of the innovation |  |
| **5. The organisations implementing the technology** |  |  |  |  |
| The organisation’s capacity to take on technological innovations is limited | - Always more complex if more than one actor is involved | - A lot of worries about new things for staff - Staff have heard other users liking the innovation - Staff are asking for the innovation without testing | - Clearly define:   - the spread of the innovation   - the rules and regulations for the innovation and its spread - create an innovation “map” |  |
| The organisation is not ready for this particular innovation | - Hierarchical structures make it difficult to implement bottom-up innovations - Inconsistent support from the region makes it difficult to manoeuvre | - The role of regional IT support is not clear and concordant in the research group | - Present the innovation to people involved in regional innovation development | - Involve the Innovation Platform as an actor in the analysis |
| Procurement processes are in place that make it more difficult to commission this technology | - The procurement of an innovation depends on whether the project is in a study phase or not - Is external money paying for the implementation of the innovation? |  | - Clearly define the phase the innovation is in |  |
| The work needed to introduce and routinise the innovation has been underestimated and/or inadequately resourced | - The very reason for the article/analysis - Everywhere an underestimation of difficulty |  | - Clearly define and present the complexities in the innovation process to the supporting organisation |  |
| **6.The external context for innovation** |  |  |  |  |
| The political and/or policy climate is adverse | - Small bottom-up innovations are at risk when implementing new major changes (i.e. new EHR system) - Is the innovation within or outside the organisation? - External changes are somewhat difficult in context | - Is the policy changing as a result of the political visions to digitalise? | - Small bottom-up innovations are at risk when implementing new major changes (new EHR system) |  |
| **7. Emergence over time** |  | - Absolute within three to five years? - Based on the actual scope or based on the desired development and implementation of the innovation? |  |  |

Note: The most useful findings, from the CCA, were that each of the four innovators needed to clarify and/or consider the following points in their own NASSS-CAT analysis:

1. The scope analysed (is the intention with the project as described in the project plan?)
2. The time frame analysed
3. The intended users “at the time of the studied project”
4. The intended adopters
5. To re-think the way value proposition can be measured
6. To consider: a) ownership, b) supply chains and c) use and spread at local, regional, national, international level belongs to both “Domain “5, organisation” and to “Domain 2, technology”
7. If the project leader changed approaches (in points 1-4) when performing the NASSS-CAT analysis, it was OK to adjust the NASSS-CAT analysis based on the new approach
8. All domains had disagreements that rendered discussions that led to negotiations
