# Supplementary material for: Exploring the Role of Complexity in Health Care Technology Bottom-Up Innovations: Multiple-Case Study Using the Nonadoption, Abandonment, Scale-Up, Spread, and Sustainability Complexity Assessment Tool
Source: JMIR Hum Factors. 2024 Apr 26;11:e50889. doi: 10.2196/50889 (PMC11087855; doi:10.2196/50889)
Supplement: Multimedia Appendix 3 [file humanfactors_v11i1e50889_app3.docx]

| **Domain** | **Issues** | **Digi-Do** | **MoodMapper** | **D-Foot** | **Point-of-care dashboard (POC)** |
| --- | --- | --- | --- | --- | --- |
| **1. THE CONDITION OR ILLNESS** | *There are significant uncertainties about the condition or illness* | Disagree | Agree | Agree | Agree |
|  | *Many people with the condition have other co-existing illnesses or impairments that affect their ability to benefit from the technology or service* | Disagree | Agree | Agree | Agree |
|  | *Many people with the condition have social or cultural factors that affect their ability to benefit from the technology or service* | Disagree | Agree | Agree | Agree |
|  | *The population with the condition, and/or how the condition is treated, is likely to change significantly over the next 3–5 years* | Disagree | Disagree | Disagree | Disagree |
|  | *The condition or illness has significant complexity which is likely to affect the success of the project* | **-** | **Yes** | **Yes** | **Yes** |
| **2. THE INNOVATION/ TECHNOLOGY** | *There are significant uncertainties about what the technology is* | Disagree | Agree | Disagree | Agree |
|  | *There are significant uncertainties about where the technology will come from* | Disagree | Agree | Agree | Agree |
|  | *There are significant uncertainties about the technology’s performance and dependability* | Disagree | Agree | Agree | Agree |
|  | *There are significant uncertainties about the technology’s usability and acceptability* | Agree | Agree | Disagree | Disagree |
|  | *There are significant technical interdependencies* | Agree | Agree | Agree | Agree |
|  | *The technology appears to be ‘disruptive’ (i.e., is likely to require major changes to organisational tasks and routines)* | Disagree | Agree | Agree | Disagree |
|  | *The technology (and/or the service model it supports) is likely to become obsolete or require replacing within the next 3–5 years* | Disagree | Disagree | Agree | Agree |
|  | *The innovation or technology has significant complexity which is likely to affect the success of the project* | **Yes** | **Yes** | **Yes** | **Yes** |
| **3. THE VALUE PROPOSITION** | *The commercial value of the technology is uncertain* | Agree | Agree | Agree | Agree |
|  | *The value to the patient or client is uncertain* | Agree | Agree | Disagree | Disagree |
|  | *The value to the clinician or other staff member is uncertain* | Disagree | Agree | Agree | Disagree |
|  | *The value to the healthcare system is uncertain* | Agree | Agree | Agree | Agree |
|  | *The value to this particular healthcare organisation is uncertain* | Disagree | Agree | Agree | Agree |
|  | *The technology could generate a negative value (costs will be more than gains) for some stakeholders* | Disagree | Disagree | Disagree | Not applicable/ Don’t know |
|  | *The value proposition is likely to change over the next 3–5 years* | Agree | Disagree | Agree | Agree |
|  | *The value proposition has significant complexity which is likely to affect the success of the project* | **Yes** | **Yes** | **Yes** | **Yes** |
| **4. THE INTENDED ADOPTERS** | *There is uncertainty about whether and how patients and their carers will adopt the technology* | Disagree | Not applicable/Don’t know | Agree | Disagree |
|  | *There is uncertainty about whether and how front-line staff will adopt the technology* | Agree | Agree | Agree | Disagree |
|  | *There is uncertainty about the implications for people indirectly affected by the technology* | Disagree | Disagree | Agree | Not applicable/Don’t know |
|  | *There will be significant changes to individual users’ perceptions of the technology over the next 3–5 years* | Disagree | Not applicable/Don’t know | Agree | Not applicable/Don’t know |
|  | *There is significant complexity relating to the intended adopters which is likely to affect the success of the project* | **Yes** | **Yes** | **Yes** | **-** |
| **5. THE ORGANISATION(S)** | *The organisation’s capacity to take on technological innovations is limited* | Agree | Agree | Agree | Agree |
|  | *The organisation is not ready for this particular innovation* | Disagree | Agree | Agree | Disagree |
|  | *Organisational routines and processes will need to change very considerably to accommodate the technology* | Disagree | Disagree | Agree | Disagree |
|  | *Procurement processes are in place that make it harder to commission this technology* | Disagree | Agree | Agree | Not applicable/Don’t know |
|  | *The work needed to introduce and routinise the innovation has been underestimated and/or inadequately resourced* | Disagree | Agree | Agree | Agree |
|  | *The organisation(s) involved are likely to have significant restructurings or changes in leadership, mission, or strategy over the next 3–5 years.* | Disagree | Disagree | Agree | Disagree |
|  | *There is significant complexity relating to one or more participating organisations which is likely to affect the success of the project* | **Yes** | **Yes** | **Yes** | **Yes** |
| **6. THE EXTERNAL CONTEXT** | *The political and/or policy climate is adverse* | Disagree | Not applicable/Don’t know | Agree | Agree |
|  | *Professional organisations are opposed to the innovation or fail to support it* | Disagree | Not applicable/Don’t know | Agree | Disagree |
|  | *Patient organisations and lobbying groups are opposed to the innovation or fail to support it* | Disagree | Disagree | Disagree | Disagree |
|  | *The regulatory context is adverse* | Agree | Not applicable/Don’t know | Agree | Disagree |
|  | *The commercial context is adverse* | Disagree | Disagree | Agree | Not applicable/Don’t know |
|  | *Opportunities for learning from other organisations are limited* | Agree | Not applicable/Don’t know | Agree | Agree |
|  | *Introduction of the technology/innovation could be threatened by external changes that impact on the organisation.* | Agree | Disagree | Agree | Agree |
|  | *The policy, regulatory and economic context for this innovation is likely to be turbulent over the next 3–5 years* | Agree | Agree | Agree | Not applicable/Don’t know |
|  | *There is significant complexity relating to the external context which is likely to affect the success of the project* | **Yes** | **Yes** | **Yes** | **Yes** |
| **7. EMERGENCE OVER TIME** | *The population with the condition, and/or how the condition is treated, is likely to change significantly over the next 3–5 years* | Disagree | Disagree | Disagree | Disagree |
|  | *The technology (and/or the service model it supports) is likely to become obsolete or require replacing within the next 3–5 years* | Disagree | Disagree | Agree | Agree |
|  | *The value proposition is likely to change over the next 3–5 years* | Agree | Disagree | Agree | Agree |
|  | *There will be significant changes to individual users’ perceptions of the technology over the next 3–5 years* | Disagree | Not applicable/Don’t know | Agree | Not applicable/Don’t know |
|  | *The organisation(s) involved are likely to have significant restructurings or changes in leadership, mission, or strategy over the next 3–5 years.* | Disagree | Disagree | Agree | Disagree |
|  | *The policy, regulatory and economic context for this innovation is likely to be turbulent over the next –5 years* | Agree | Agree | Agree | Not applicable/Don’t know |

*Note: The orange agreement fields indicate when the innovation is judged to be complex or not (green colour)*
